# Improving the Stability of Ball-Milled Lead Halide Perovskites via Ethanol/Water-Induced Phase Transition

**DOI:** 10.3390/nano12060920

**Published:** 2022-03-10

**Authors:** Jinyoung Kim, Nguyen The Manh, Huynh Tan Thai, Soon-Ki Jeong, Young-Woo Lee, Younghyun Cho, Wook Ahn, Yura Choi, Namchul Cho

**Affiliations:** Department of Energy Systems Engineering, Soonchunhyang University, 22 Soonchunhyang-ro, Shinchang-myeon, Asan-si 31538, Chungcheongnam-do, Korea; thn05007@gmail.com (J.K.); manh01687589807@gmail.com (N.T.M.); huynhtanthai03@gmail.com (H.T.T.); hamin611@sch.ac.kr (S.-K.J.); ywlee@sch.ac.kr (Y.-W.L.); yhcho@sch.ac.kr (Y.C.); wahn21@sch.ac.kr (W.A.); bnbn3238@sch.ac (Y.C.)

**Keywords:** CsPbBr_3_ perovskite nanocrystals, ball milling, phase transition, stability, mass production

## Abstract

Recently, lead halide perovskite nanocrystals have been considered as potential light-emitting materials because of their narrow full width at half-maximum (FWHM) and high photoluminescence quantum yield (PLQY). In addition, they have various emission spectra because the bandgap can be easily tuned by changing the size of the nanocrystals and their chemical composition. However, these perovskite materials have poor long-term stability due to their sensitivity to moisture. Thus far, various approaches have been attempted to enhance the stability of the perovskite nanocrystals. However, the required level of stability in the mass production process of perovskite nanocrystals under ambient conditions has not been secured. In this work, we developed a facile two-step ball-milling and ethanol/water-induced phase transition method to synthesize stable CsPbBr_3_ perovskite materials. We obtained pure CsPbBr_3_ perovskite solutions with stability retention of 86% for 30 days under ambient conditions. Our materials show a high PLQY of 35% in solid films, and excellent thermal stability up to 80 °C. We believe that our new synthetic method could be applicable for the mass production of light-emitting perovskite materials.

## 1. Introduction

Recently, lead halide perovskites (LHPs) have become the ideal candidate materials for solar cells [1,2,3], light-emitting diodes [2,4,5,6,7], photodetectors [8,9,10], X-ray imaging [11,12,13,14], and lasers [2,15]. This is because of the remarkable photophysical properties of LHPs, such as adjustable bandgaps, high photoluminescence quantum yield (PLQY) [16], narrow full width at half-maximum (FWHM) [17,18], and defect tolerance. However, the ionic nature of LHPs, along with degradation caused by moisture, polar solvents, and light, results in poor stability.

The synthesis of CsPbX_3_ (X = Cl, Br, I) nanocrystals (NCs) using a hot-injection method was first reported in 2015. Consequently, the PLQY of the solution state reached 90% [19]. Since then, researchers have increased the stability and PLQY via surface modifications [18,20,21,22] and encapsulation with hydrophobic polymer matrices [23,24]. The hot-injection method requires a high reaction temperature and an inert environment [16,25,26]. Therefore, this method has high mass-production costs. In the hot-injection method, the reaction temperature is related to the NC size. During synthesis, a precursor solution is injected into the reaction chamber. Generally, the temperature of the precursor solution is lower than the reaction temperature, making it difficult to control the reaction temperature. Therefore, this hot-injection method is not suitable for mass production [27].

As an alternative, the ligand-assisted reprecipitation (LARP) method has been reported by several researchers [27]. This method can be performed at room temperature under ambient conditions using polar solvents. Because LHP NCs can be decomposed in polar solvents, the LARP method is not suitable for mass production [28].

Li et al. reported the synthesis of CsPbBr_3_ NCs using the supersaturated recrystallization (SR) method [29]. This method is based on mixing solvents with different polarities. The method is performed by transferring Cs^+^, Pb^2+^, and Br^−^ ions from soluble solvents to insoluble solvents. This ion transfer leads to a supersaturated state, allowing the formation of CsPbBr_3_ perovskite NCs in the presence of ligands. Similar to the LARP method, the SR method is also performed at room temperature under ambient conditions. However, this method also uses polar solvents, which can cause the instability of perovskite NCs.

The ball-milling method is a useful method for synthesizing LHP NCs [30]. Mukasyan et al. reported the synthesis of methylammonium lead iodide perovskite (CH_3_NH_3_PbI_3_) by ball milling [31]. Zhu et al. developed a simple solvent-free system to obtain highly emissive cesium lead halide perovskite (CsPbX_3_) quantum dots via ball milling [32]. Jiang et al. proposed double ligands for improving the PLQY by using partially hydrolyzed poly(methyl methacrylate) and highly branched poly(ethylenimine) via mechanochemical synthesis. Consequently, the CsPbBr_3_/Cs_2_PbBr_5_ NCs exhibited good photostability and high chemical stability [23]. Protescu et al. reported a low-cost wet ball-milling method for a mixture of bulk APbBr_3_ (A = Cs, FA), solvents, and ligands [32]. These studies demonstrated that the ball-milling method is suitable for producing stable LHP NCs in large-scale reactions. More importantly, the ball-milling method is free from temperature control, which causes high manufacturing costs. In addition, the ball-milling method is free from using polar solvents; therefore, it is more suitable for the mass production of perovskite NCs than the conventional synthetic methods.

Although the ball-milling method is suitable for the mass production of perovskite, it has several disadvantages. For example, the reaction proceeds in air, and mechanical grinding can cause many defects on the surface. Surface defects generated by grinding can seriously impair luminescent properties; therefore, it is very important to find a way to overcome these problems. Recently, Wu et al. reported that the concentration of surface defects in CsPbBr_3_ NCs was significantly reduced when they were synthesized via the water-induced phase transition method [33]. The authors synthesized Cs_4_PbBr_6_ NCs that undergo a phase transition, via hot injection. However, we attempted to obtain CsPbBr_3_ with reduced surface defects through a phase transition process, after obtaining Cs_4_PbBr_6_ via ball milling for mass production.

The structure and optical properties of CsPbBr_3_ synthesized via the water-induced phase transition method were studied using XRD, absorption, and photoluminescence (PL) spectroscopy. The phase-transformed CsPbBr_3_ exhibited higher PL properties than Cs_4_PbBr_6_. This study confirmed that filmed CsPbBr_3_ has a PLQY of 35%. The synthesized pure CsPbBr_3_ perovskite solution showed a higher stability (86%) than the conventional methods after 30 days under ambient conditions. We believe that the increased thermal stability originated from the decreased defect density achieved on the surface of the perovskite microcrystals by introducing a polymer ligand. Because the produced LHP exhibited high PL intensity and stability, it shows strong application potential in the lighting and display fields.

## 2. Materials and Methods

### 2.1. Materials

Cesium(I) bromide (CsBr; 99.999%), lead(II) bromide (PbBr_2_; 99.999%), oleylamine (OAm; technical grade 70%), octadecene (ODE; technical grade 90%), and poly(acrylic acid) (PAA; average Mw = 1800) were purchased from Sigma-Aldrich (St. Louis, MO, USA). Toluene (≥99.5%) and ethanol (95%) were purchased from Samchun Chemical (Seoul, Korea). Oleic acid (OA; ≥85%) was purchased from Tokyo Chemical Industry (Tokyo, Japan). Hydrobromic acid (HBr; 47%) was purchased from Tokyo Chemical Industry.

### 2.2. Methods

#### 2.2.1. Preparation of Cs_4_PbBr_6_ Crystal

Bulk Cs_4_PbBr_6_ crystals were synthesized using a previously reported method [34]. First, 0.76 g of CsBr (0.2 M) and 0.32 g of PbBr_2_ (0.05 M) were dissolved in 18 mL of dimethyl sulfoxide (DMSO) solvent under ultrasonication. After complete dissolution, the precursor solution was filtered through a syringe filter, and then injected into a 20 mL glass vial. The glass vial was sealed with aluminum foil with a small hole in it, and then was stored in a 250 mL glass container under a dichloromethane (DCM) atmosphere at room temperature. Before closing the container, 1.5 mL of HBr was quickly injected into the precursor solution. The suitable time to complete the crystallization process (obtain crystals) was 4 days. The crystals were carefully washed with DMSO solution twice and, finally, washed with DCM. After purification, the crystals were dried under ambient conditions.

#### 2.2.2. Mechanochemical Synthesis of Cs_4_PbBr_6_ Microcrystals (MCs)

Bulk Cs_4_PbBr_6_ (0.15 g), OAm (300 μL), toluene (3 mL), and PAA (25 mg) were loaded into ball-milling jars, and were ground for 2 h at 400 rpm (Figure S1). Next, 17 mL of toluene was added to dilute the mixture.

#### 2.2.3. Synthesis of Ethanol/Water-Induced CsPbBr_3_ MCs

The transformation process in this experiment was conducted under ambient conditions. Two milliliters of the as-prepared Cs_4_PbBr_6_ MC solutions were injected into vials. Ethanol/water (200 µL, at a ratio of 4:1) was added to the solutions sequentially. Each vial was diluted with 6 mL of toluene to obtain MCs. After 4 days, the Cs_4_PbBr_6_ MCs completely transformed into CsPbBr_3_ MCs. Finally, high-speed centrifugation at 4000 rpm for 3 min was used to collect the supernatant for further characterization.

#### 2.2.4. Synthesis of CsPbBr_3_ MCs through the Hot-Injection Method

To synthesize cesium oleate, 0.41 g of Cs_2_CO_3_ was mixed with 1.25 mL of OA and 20 mL of ODE, which were loaded into a round flask and dried at 120 °C, and then annealed in a nitrogen atmosphere for 40 min before annealing at 150 °C for a further 40 min. Subsequently, PbBr_2_ (70 mg), OA (0.5 mL), OAm (0.5 mL), and ODE (5 mL) were loaded into a 25 mL round flask. The mixture was annealed at 120 °C for 40 min under vacuum, and then the temperature was increased to 170 °C for 30 min. Finally, the cesium oleate (0.4 mL) was injected into the g immersed in an ice-water bath. For purification, the crude solution containing ethyl acetate was centrifuged at 8000 rpm for 4 min. After centrifugation, the CsPbBr_3_ MCs were dispersed in toluene.

### 2.3. Structural and Optical Characterization

The absorption and PL spectra of the colloidal solutions were recorded using a FluoroMax-4 spectrofluorometer (Horiba Jobin Yvon, Kyoto, Japan). X-ray diffraction (XRD) spectra were obtained using a Rigaku, MiniFlex diffractometer (Tokyo, Japan) in the 2θ range of 10–50°. The morphology of the perovskite thin film before and after the phase transition process was examined via scanning electron microscopy (SEM; Apreo S HiVac, FEI, Hillsboro, OR, USA). The PLQY of the thin film was recorded using an integrating sphere (Figure S2) with a FluoroMax-4 spectrofluorometer (Horiba Jobin Yvon). The film was prepared on bare glass (15 mm × 15 mm).

## 3. Results and Discussion

### 3.1. Synthesis of CsPbBr_3_ MCs

**Figure 1:**
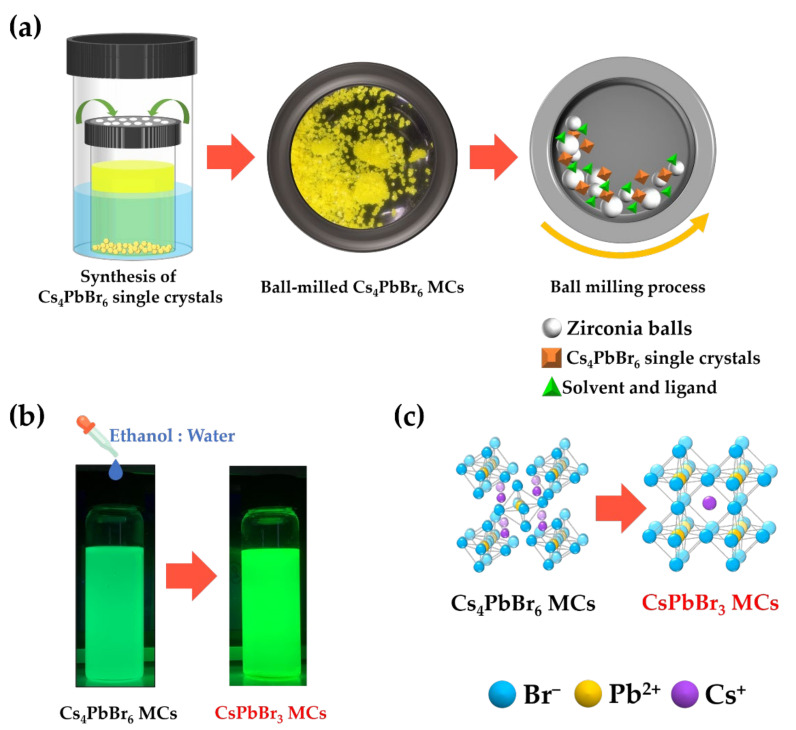
Schematic diagram of the phase transition process from Cs_4_PbBr_6_ MCs to CsPbBr_3_ using an ethanol/water treatment: (**a**) process to obtain Cs_4_PbBr_6_ MCs via the ball-milling process, (**b**) photographic image of the Cs_4_PbBr_6_ and CsPbBr_3_ solutions under UV light (365 nm) with an ethanol/water treatment, and (**c**) crystal structure of the Cs_4_PbBr_6_ and CsPbBr_3_ MCs.

Figure 1 shows the process of the phase transition from Cs_4_PbBr_6_ to CsPbBr_3_ MCs through the ethanol/water treatment. The bulk Cs_4_PbBr_6_ single crystals were synthesized and purified using previously reported methods [34]. Then, the bulk Cs_4_PbBr_6_ single crystals were ball-milled with a ligand (PAA) and solvent (toluene) to obtain Cs_4_PbBr_6_ MCs. After adding 200 µL of an ethanol/water cosolvent to the synthesized Cs_4_PbBr_6_ MC solution, the crystals were allowed to undergo a phase transition process for 4 days to obtain CsPbBr_3_ MCs. The Cs_4_PbBr_6_ solution exhibited a bright green PL after the phase transition. The detailed phase transition process was further analyzed by measuring the absorption, PL, and XRD spectra.

### 3.2. XRD Analysis

**Figure 2:**
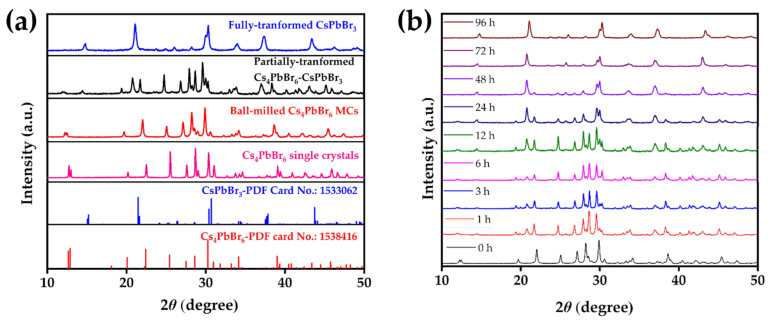
(**a**) XRD spectra of the synthesized bulk Cs_4_PbBr_6_ single crystals, Cs_4_PbBr_6_ MCs obtained through ball milling, and phase-transformed CsPbBr_3_ MCs. The standard XRD spectra of rhombohedral Cs_4_PbBr_6_ and orthorhombic CsPbBr_3_ are also indicated. (**b**) XRD spectra indicating the phase transition of ethanol/water-treated Cs_4_PbBr_6_ MCs to CsPbBr_3_ MCs with differing times.

The XRD patterns of the synthesized Cs_4_PbBr_6_ single crystals, Cs_4_PbBr_6_ MCs, and CsPbBr_3_ MCs with rhombohedral Cs_4_PbBr_6_ and orthorhombic CsPbBr_3_ crystal structures were observed at each step (Figure 2a). The XRD patterns of the Cs_4_PbBr_6_ single crystals and ball-milled Cs_4_PbBr_6_ MCs were similar. The black line in Figure 2 represents the XRD spectrum of the partially transformed Cs_4_PbBr_6_ MCs, showing a clear (012) peak. However, the blue line in Figure 2 represents the XRD spectrum of the fully transformed CsPbBr_3_ MCs, which did not show a (012) peak. This indicates a complete phase transition from Cs_4_PbBr_6_ MCs to CsPbBr_3_ MCs. Figure 2b shows the gradual change in the XRD patterns due to the phase transition process caused by the ethanol/water treatment. Note that the (012) peak of the XRD spectrum of the Cs_4_PbBr_6_ MCs disappeared with increasing reaction time. However, the (100) peak of the XRD spectrum of the CsPbBr_3_ MCs appeared as the reaction time progressed. The (012) XRD peak of the Cs_4_PbBr_6_ MCs completely disappeared after 96 h.

### 3.3. Absorption and Emission Spectroscopy

**Figure 3:**
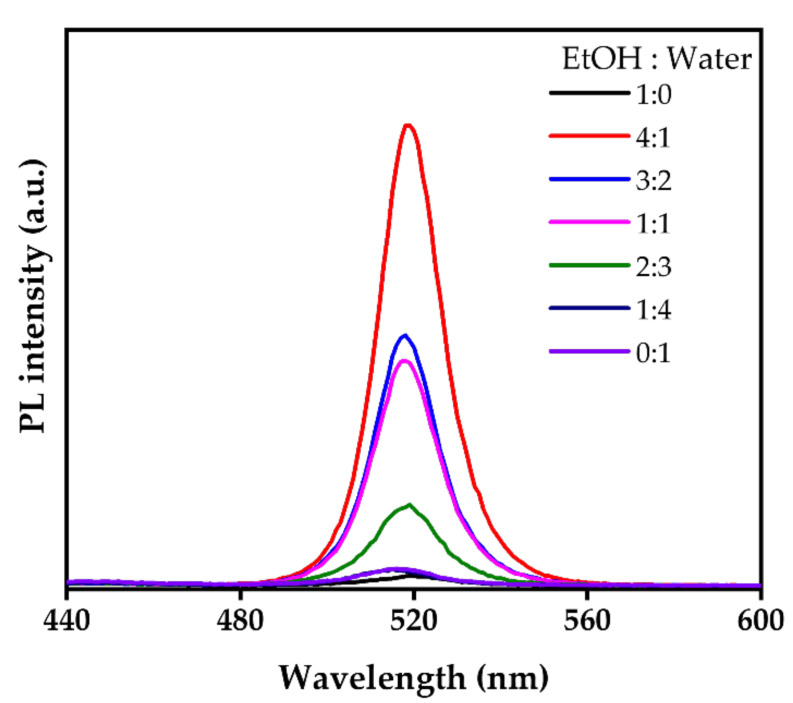
Comparison of the PL intensity of the CsPbBr_3_ MC solutions obtained via the phase transition process with different ratios of ethanol/water.

Figure 3 shows the PL intensities of the CsPbBr_3_ MC solutions obtained via the phase transition process with different ratios of ethanol/water. The maximum PL intensity was observed at an ethanol/water ratio of 4:1. The lowest PL intensity was observed when ethanol or water was used alone. Recently, Wu et al. reported that the phase transition process occurs when CsBr is stripped from the Cs_4_PbBr_6_ NCs by a polar solvent such as water [33]. Turedi et al. also reported that CsPbBr_3_ films undergo a phase transition to stable two-dimensional (2D) CsPb_2_Br_5_ through water treatment [35]. The detailed mechanism of the transition process is discussed further below.

**Figure 4:**
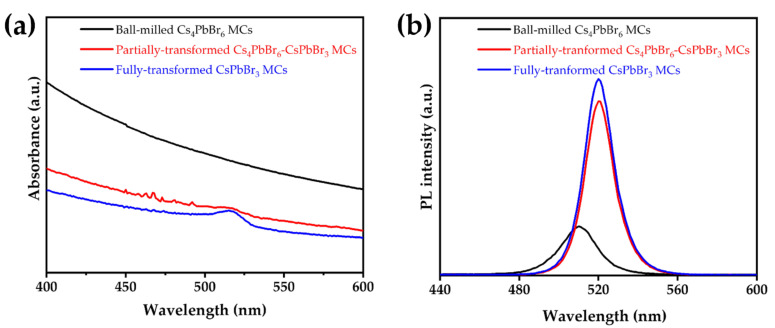
(**a**) Absorption and (**b**) PL spectra of the ball-milled Cs_4_PbBr_6_ MCs, partially transformed Cs_4_PbBr_6_–CsPbBr_3_ MCs, and fully transformed CsPbBr_3_ MCs.

The absorption and PL spectra show the phase transition process from Cs_4_PbBr_6_ MCs to CsPbBr_3_ MCs through an ethanol/water treatment (Figure 4). No peaks at 520 nm were observed in the absorption spectrum of the ball-milled Cs_4_PbBr_6_ MCs. However, a slight peak at 520 nm emerged in the absorption spectrum of the partially transformed Cs_4_PbBr_6_–CsPbBr_3_ MCs. Moreover, a pronounced absorption peak at 520 nm was observed in the absorption spectrum of the fully transformed CsPbBr_3_ MCs. In the PL spectra, the Cs_4_PbBr_6_ MCs exhibited the lowest PL intensity and an FWHM of 20 nm at 511 nm. The partially transformed Cs_4_PbBr_6_–CsPbBr_3_ MCs exhibited a higher PL intensity and narrower FWHM than the Cs_4_PbBr_6_ MCs. The CsPbBr_3_ MCs exhibited the highest PL intensity and an FWHM of 17 nm at 520 nm. The improvement in PL intensity and narrowing of the FWHM was due to the decrease in the defect density on the surface of the perovskite microcrystals caused by the ethanol/water treatment. The bandgaps for Cs_4_PbBr_6_ and CsPbBr_3_ were estimated to be approximately 3.70 and 2.25 eV, respectively (Figure S3), which are in good agreement with previously reported values [36,37,38]. Wu et al. reported that a nonpolar solvent with low miscibility in water was used to prevent the decomposition of the obtained CsPbBr_3_ NCs by water.

This study employed a polymer ligand, instead of a nonpolar solvent, which can improve the optical properties and stability of polar solvents. Polymer ligands are known to prevent the degradation of perovskite MCs by polar solvents [33]. Furthermore, the polymer ligand decreases the non-radiative recombination rate of charge carriers, thereby increasing the PLQY [39].

**Figure 5:**
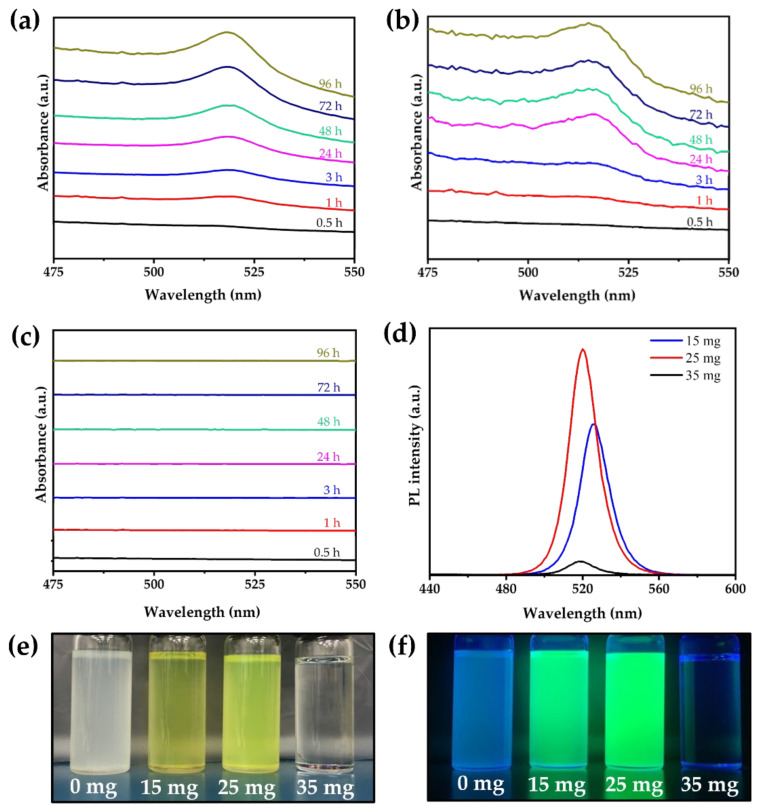
Time-dependent absorption spectra of the CsPbBr_3_ MC solutions synthesized using different amounts of PAA: (**a**) 15 mg, (**b**) 25 mg, and (**c**) 35 mg PAA. (**d**) PL spectra of the CsPbBr_3_ MC solutions synthesized with different amounts of PAA obtained via the ethanol/water treatment. Photographic images of the CsPbBr_3_ MC solutions (**e**) under room light and (**f**) UV light.

The absorption spectra of the CsPbBr_3_ MC solutions prepared with different amounts of PAA as the polymer ligand were obtained (Figure 5a–c). When 15 and 25 mg of PAA were used, an absorption peak of CsPbBr_3_ appeared at 520 nm, but did not appear when 35 mg of PAA was used. Since the absorption of the CsPbBr_3_ MC solutions was related to the amount of PAA, the PL spectra were further analyzed. Figure 5d shows the PL spectra of the CsPbBr_3_ MCs with different amounts of PAA. The CsPbBr_3_ MCs synthesized with 35 mg of PAA exhibited the lowest PL intensity. However, the CsPbBr_3_ MCs synthesized with 15 mg of PAA exhibited a higher PL intensity, and those synthesized with 25 mg of PAA exhibited the highest PL intensity. In Figure 5e,f, the CsPbBr_3_ MC solutions synthesized with 25 mg PAA showed the brightest green PL, and the solutions synthesized with 15 mg PAA showed a slightly weaker PL. However, the solutions synthesized with 35 mg of PAA showed the weakest PL, which was almost transparent. The PL spectra and emission from the CsPbBr_3_ MC solutions under visible and ultraviolet light revealed that the CsPbBr_3_ MCs synthesized with 25 mg of PAA had the best emission properties. Additionally, the CsPbBr_3_ MC solution, which was not synthesized with PAA, was degraded by ethanol and water.

Generally, the chemical structure of the ligands affects the optical properties and film morphology of the perovskite NCs [40,41,42,43]. Sichert et al. reported that the nanoplatelet thickness could be controlled by the amount of OAm, which could affect the quantum size effect in two-dimensional perovskite [40]. Jiang et al. reported various optical properties depending on the amount of polymer, which improved the stability by forming a shell on the perovskite surface [42]. It has also been reported that the optical properties of perovskite can be significantly decreased by side reactions when polymers or monomolecular ligands are used in excess amounts [42,43]. Inferior optical properties were also observed when 35 mg of PAA was used to make CsPbBr_3_ MC solutions, which could be the result of using an excessive amount of polymer ligand.

**Figure 6:**
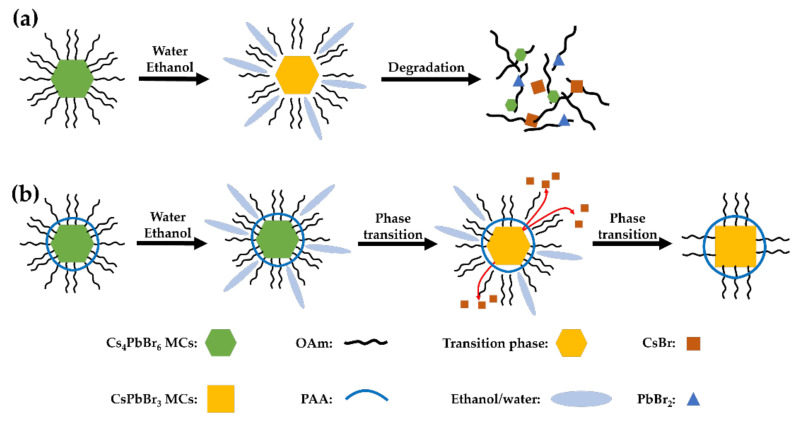
Schematic illustration of the phase transition process of Cs_4_PbBr_6_ MCs obtained via ethanol/water treatment (**a**) without PAA and (**b**) with PAA.

Figure 6 shows the phase transition process of the Cs_4_PbBr_6_ MCs with and without PAA. Cs_4_PbBr_6_ MCs, which are not synthesized with PAA and only use OAm as a ligand, are easily degraded by ethanol and water (Figure 6a) [39]. Cs_4_PbBr_6_ MCs synthesized with PAA undergo a phase transition process without degradation, because PAA protects them from polar solvents (Figure 6b). PAA is a polymer chain with a carboxyl group, and since the carboxyl group surrounds the Cs_4_PbBr_6_ MC surface and bonds with the lead on the surface, it can form a stronger bond than OAm [23]. Therefore, when Cs_4_PbBr_6_ MCs capped with PAA and OAm are treated with ethanol/water, OAm dissociates and CsBr is stripped by ethanol and water, so it undergoes a phase transition [44].

### 3.4. Phase Transformation Conditions and Mechanisms

**Figure 7:**
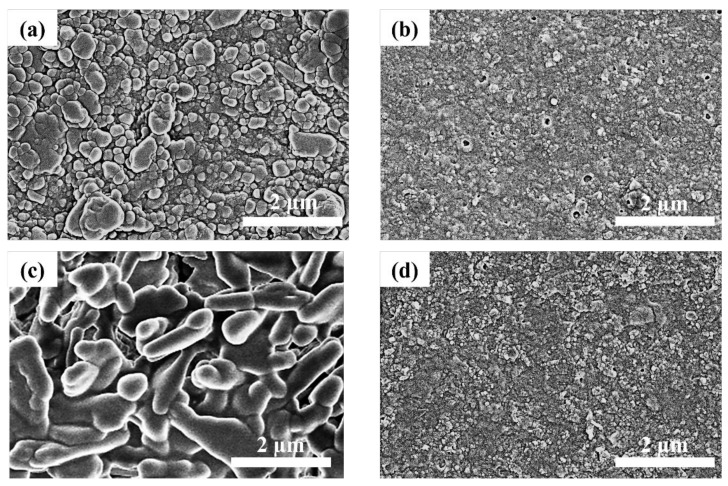
SEM images of the Cs_4_PbBr_6_ MC films before ethanol/water treatment (**a**) without PAA and (**b**) with PAA. SEM images of the CsPbBr_3_ MC films after ethanol/water treatment (**c**) without PAA and (**d**) with PAA.

The morphologies of Cs_4_PbBr_6_ and CsPbBr_3_ MCs with and without PAA were characterized using SEM (Figure 7). Before the ethanol/water treatment, the grains of the Cs_4_PbBr_6_ MCs synthesized without PAA were large. However, the grains of the Cs_4_PbBr_6_ MCs synthesized with PAA were small and regular. This is because OAm, used as a ligand, can contribute to size control in the ball-milling process [45]. After the ethanol/water treatment, the grains of Cs_4_PbBr_6_ MCs synthesized without PAA disappeared, and the Cs_4_PbBr_6_ MCs synthesized with PAA had relatively small grains. This indicates that PAA can prevent the degradation of Cs_4_PbBr_6_ MCs in ethanol and water.

### 3.5. Stability of Ethanol/Water-Induced CsPbBr_3_ MCs

**Figure 8:**
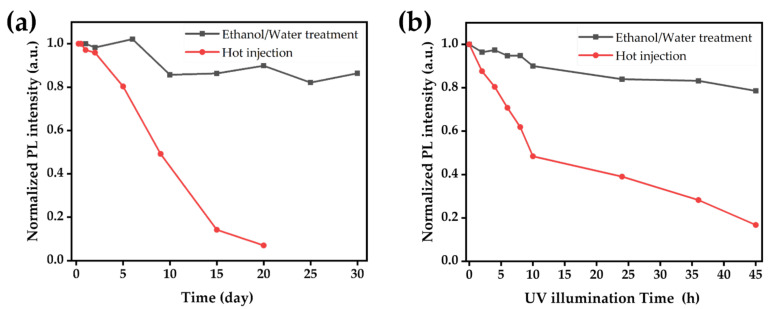
(**a**) Time-dependent PL spectra of the CsPbBr_3_ MC solution obtained via the ethanol/water treatment and hot-injection method under ambient conditions, and (**b**) PL spectra of the CsPbBr_3_ MC solution obtained via the ethanol/water treatment and high-temperature injection under UV Illumination.

The stability of the CsPbBr_3_ MCs treated with ethanol/water was compared to that of the OAm/OA–CsPbBr_3_ MCs under ambient conditions and UV light. OAm/OA–CsPbBr_3_ MCs were synthesized using a hot-injection method. The XRD, absorption, and PL spectra were measured to confirm the crystal structure and optical properties of the OAm/OA-capped CsPbBr_3_ MCs. (Figure S4). The PL spectra in Figure 8a show a difference in stability between the OAm/OA-capped CsPbBr_3_ MCs and the CsPbBr_3_ MCs under ambient conditions. The PL intensity of OAm/OA-capped CsPbBr_3_ MCs synthesized by hot injection decreased to 7% of the initial PL intensity after 20 days. However, the CsPbBr_3_ MCs synthesized in this study only decreased to 86% of their initial PL intensity, even after 30 days. The difference in stability between the OAm/OA-capped CsPbBr_3_ MCs and the CsPbBr_3_ MCs under UV light was further examined through the PL spectra (Figure 8b). The PL intensity of the OAm/OA-capped CsPbBr_3_ MCs synthesized by hot injection decreased to 28% of their initial PL intensity after 45 h. However, the CsPbBr_3_ MCs decreased to 78% of their initial PL intensity after 45 h. The ability of the CsPbBr_3_ MCs to preserve their initial PL intensity throughout the tests outperformed that of the widely used OAm/OA-CsPbBr_3_ MCs, regardless of ambient conditions and UV irradiation.

### 3.6. Thermal Stability of the CsPbBr_3_ MC Thin Film

**Figure 9:**
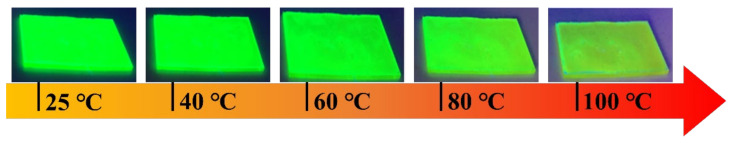
Photographic images of CsPbBr_3_ MC thin films under UV light with thermal annealing.

To fabricate thin films of CsPbBr_3_ MCs, the glass substrates were sequentially sonicated in acetone and isopropyl alcohol for 10 min. The CsPbBr_3_ MC solution was drop-casted onto the glass substrate and annealed at 70 °C for 5 min under ambient conditions. After that, we annealed each film on the preheated hot plate for 10 min, and took photographic images under UV irradiation (365 nm), as shown in Figure 9. We examined the thermal stability of CsPbBr_3_ MC films in the range of 25–100 °C for 10 min, and found that the green emission was maintained up to 80 °C, and started to decrease at 100 °C. This result indicates that the phase-transformed CsPbBr_3_ MC films have good thermal stability up to 80 °C.

The PLQY of the CsPbBr_3_ MC film was calculated from the absorption and PL spectra of the sample and reference (Figure S5). PLQY is calculated as the ratio of the amount of radiation emitted to the amount of radiation absorbed by the CsPbBr_3_ MC film (Equation (1)) [46].

(1) $$\mathrm{PLQY}=\frac{E_{\mathrm{Sample}}{-E}_{\mathrm{Ref}}}{S_{\mathrm{Ref}}{-S}_{\mathrm{Sample}}}\;$$

where *S_Ref_* and *E_Ref_* are the absorption and PL terms of the reference, respectively, and *S_Sample_* and *E_Sample_* are the absorption and PL terms of the sample, respectively. Each term corresponds to the integral value of the absorption and PL spectra. The PLQY of the CsPbBr_3_ MC film in this study was 35%. Sousa et al. reported that perovskite MCs with a poor size distribution had a lower PLQY [47]. To compare the size distribution of our MCs against others, it was calculated as a percentage of the distribution of mean size to the standard deviation. The percentage of the distribution of the mean size to the standard deviation of CsPbBr_3_ MCs in this study was 22.89% (Figure S6). This result was higher than 6.7%, which is the percentage of the distribution of mean size to the standard deviation of CsPbBr_3_ NCs using LARP [48].

## 4. Conclusions

This study proposed a method for the mass production of CsPbBr_3_ MCs with high thermal stability under ambient conditions. A facile two-step ball-milling and ethanol/water-induced phase transition method was developed to synthesize stable CsPbBr_3_ perovskite materials. PAA was used as a ligand to protect the perovskite MCs from the ethanol/water and ambient conditions throughout the reaction. The PL spectra revealed that 0.25 mg of PAA imparts the best optical properties to the CsPbBr_3_ MC solutions. Our ball-milled MC solutions showed 80% higher air stability than the hot-injection method after 30 days under ambient conditions. Additionally, our solutions showed 50% higher stability than the hot-injection method after 45 h of UV irradiation. The CsPbBr_3_ MC films also showed excellent thermal stability up to 80 °C. The relatively high solid-state PLQY (35%) originates from the surface passivation effect of the PAA ligand. We believe that our facile mass production method can pioneer the industrial application of perovskite nanomaterials.

## Data Availability

Not applicable.

## Supplementary Materials

The following supporting information can be downloaded at https://www.mdpi.com/article/10.3390/nano12060920/s1: Figure S1: XRD patterns of Cs_4_PbBr_6_ NCs as a function of ball-milling time, Figure S2: Illustration of integrating sphere, Figure S3: Tauc plot for determining the bandgaps of the (**a**) Cs_4_PbBr_6_ MCs, and (**b**) CsPbBr_3_ MCs. The band gap energy was obtained by extrapolating the straight-line portion of the graph to zero absorption coefficients. The intercept on the energy axis indicates the value of band gap energy, Figure S4: (**a**) Observed (black solid line), reference (green solid line), and difference (bottom gray line) powder X-ray diffraction (XRD) patterns for CsPbBr3, (**b**) absorption and PL spectra of CsPbBr_3_ MCs obtained through hot-injection method comparison with ethanol/water-treated CsPbBr_3_ MCs, Figure S5: (**a**) Photoluminescence spectrum and (**b**) absorption spectrum for the measurement of photoluminescence quantum yield of CsPbBr3 MCs film, Figure S6: Scanning electron microscope (SEM) image and size distribution graph of CsPbBr_3_ MCs obtained through ball milling process and ethanol/water treatment.
