# Supplementary material for: Improving the Stability of Ball-Milled Lead Halide Perovskites via Ethanol/Water-Induced Phase Transition
Source: Nanomaterials (Basel). 2022 Mar 10;12(6):920. doi: 10.3390/nano12060920 (PMC8954044; doi:10.3390/nano12060920)
Supplement: Supplementary file 1 [file nanomaterials-12-00920-s001.zip › nanomaterials-1611161-supplementary.pdf]

nanomaterials

Supplementary Materials

Improving the Stability of Ball-Milled Lead Halide Perovskites via Ethanol/Water-Induced Phase Transition

Jinyoung Kim, Nguyen The Manh, Huynh Tan Thai, Soon-Ki Jeong, Young-Woo Lee, Younghyun Cho, Wook Ahn, Yura Choi and Namchul Cho *

Department of Energy Systems Engineering, Soonchunhyang University, 22 Soonchunhyang-ro, Shinchang-myeon, Asan-si 31538, Chungcheongnam-do, Korea; thn05007@gmail.com (J.K.); manh01687589807@gmail.com (N.T.M.); huynhtanthai03@gmail.com (H.T.T.); hamin611@sch.ac.kr (S.-K.J.); ywlee@sch.ac.kr (Y.-W.L.); yhcho@sch.ac.kr (Y.C.); wahn21@sch.ac.kr (W.A.); bnbn3238@sch.ac (Y.C.)

* Correspondence: chon7@sch.ac.kr

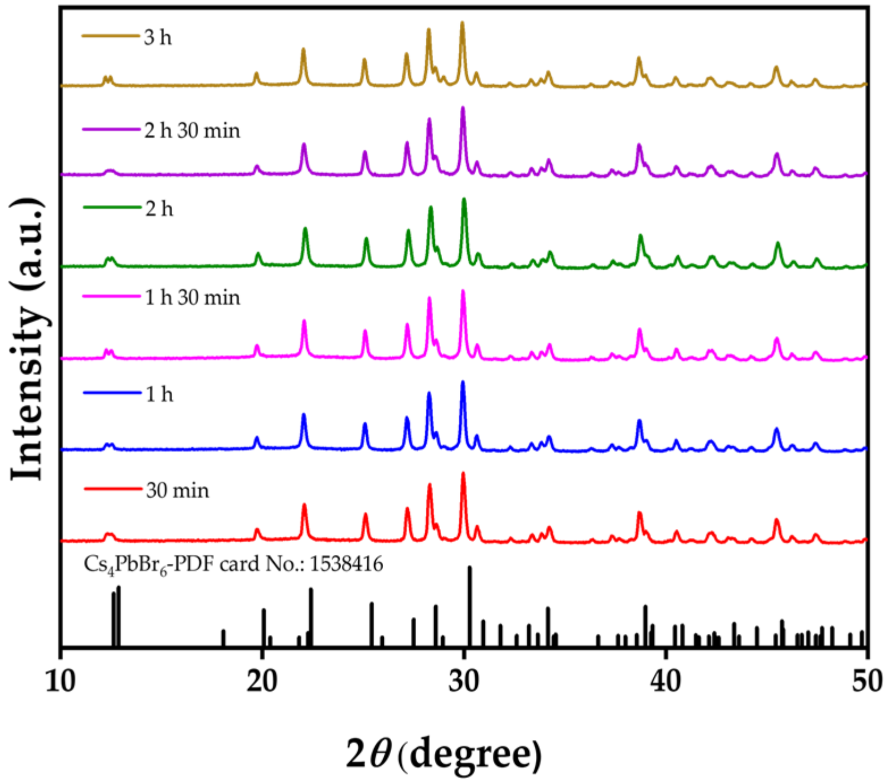

Figure S1. XRD patterns of Cs_4PbBr_6 NCs as a function of ball-milling time.

We measured ball milling time dependent XRD of Cs_4PbBr_6. The XRD data of Cs_4PbBr_6 ball milled for 30–180 min showed negligible difference. We applied 2 h of reaction time to make small and fully reacted perovskite microcrystals.

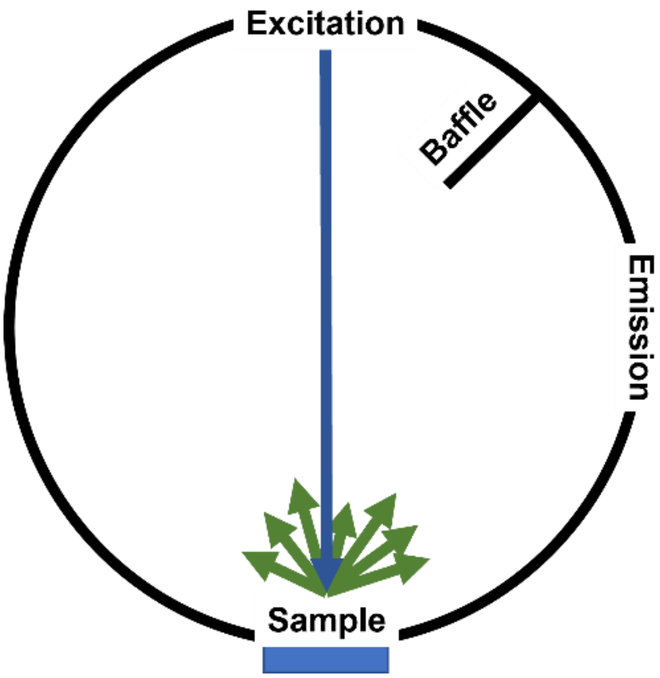

Figure S2. Illustration of integrating sphere.

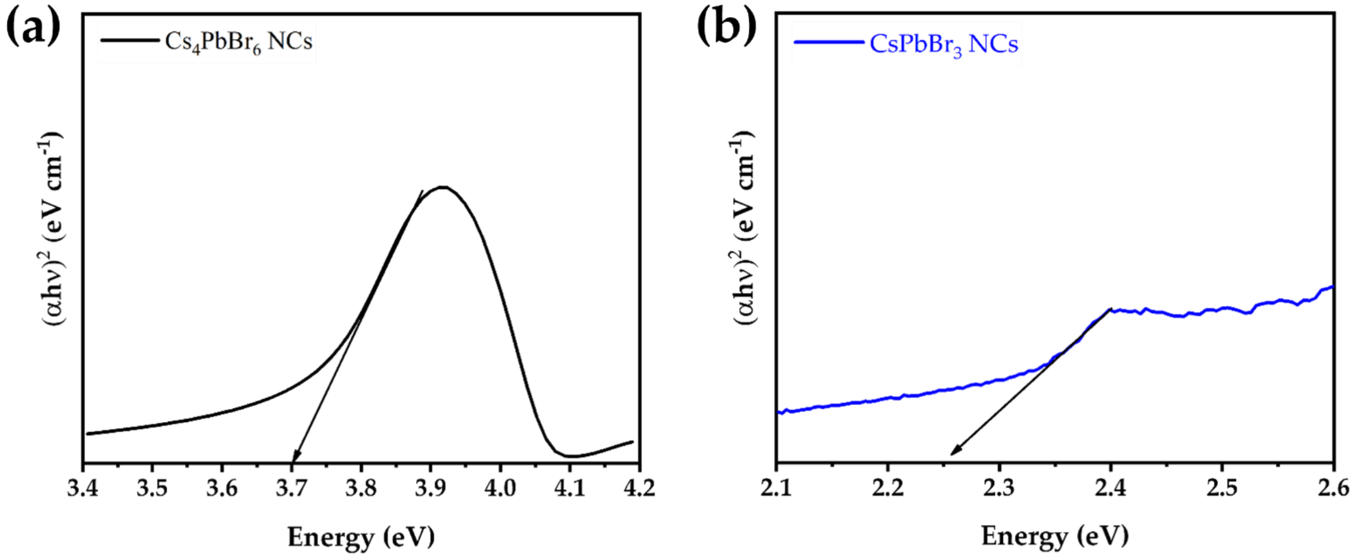

Figure S3. Tauc plot for determining the bandgaps of the (**a**) Cs_4PbBr_6 MCs, and (**b**) $CsPbBr_3$ MCs. The band gap energy was obtained by extrapolating the straight-line portion of the graph to zero absorption coefficients. The intercept on the energy axis indicates the value of band gap energy.

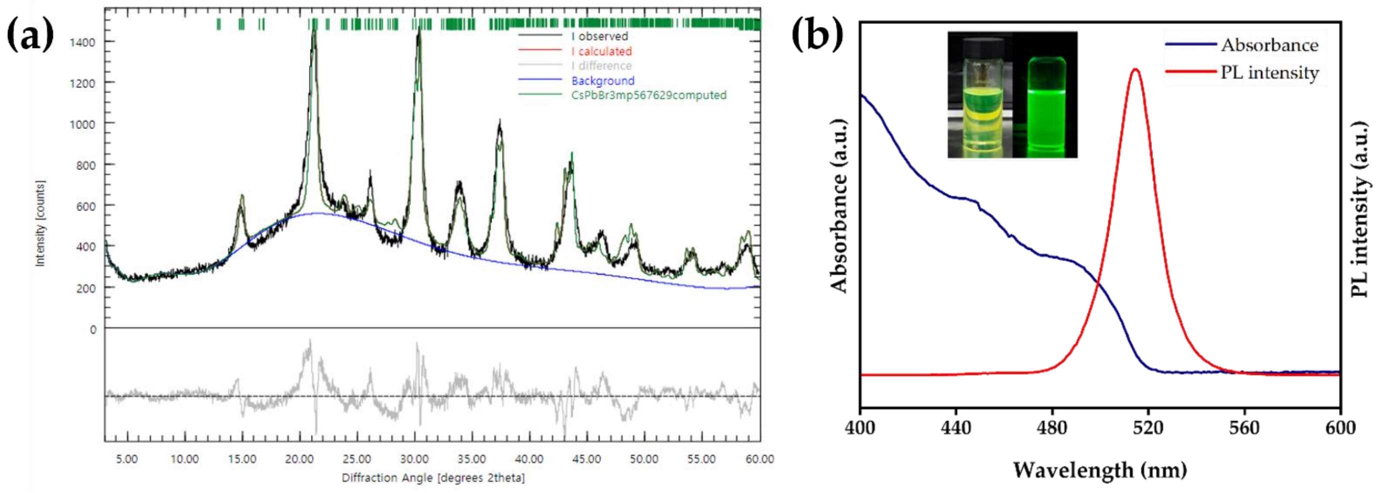

Figure S4. (**a**) Observed (black solid line), reference (green solid line), and difference (bottom gray line) powder X-ray diffraction (XRD) patterns for CsPbBr3, (**b**) absorption and PL spectra of $CsPbBr_3$ MCs obtained through hot-injection method comparison with ethanol/water-treated $CsPbBr_3$ MCs.

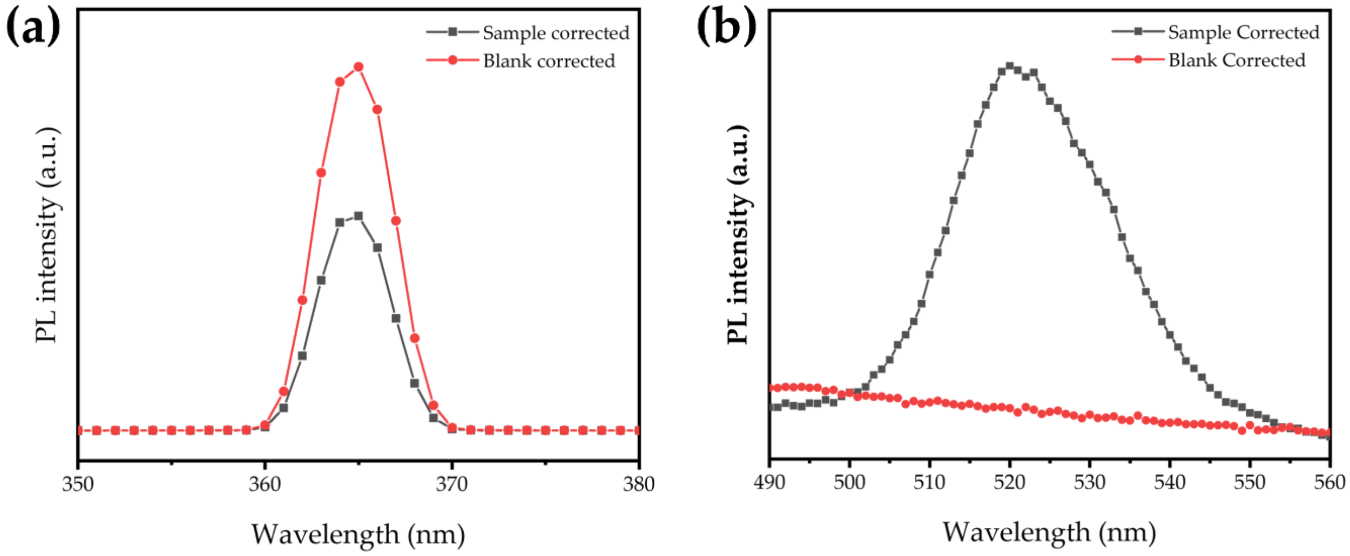

Figure S5. (**a**) Photoluminescence spectrum and (**b**) absorption spectrum for the measurement of photoluminescence quantum yield of CsPbBr3 MCs film.

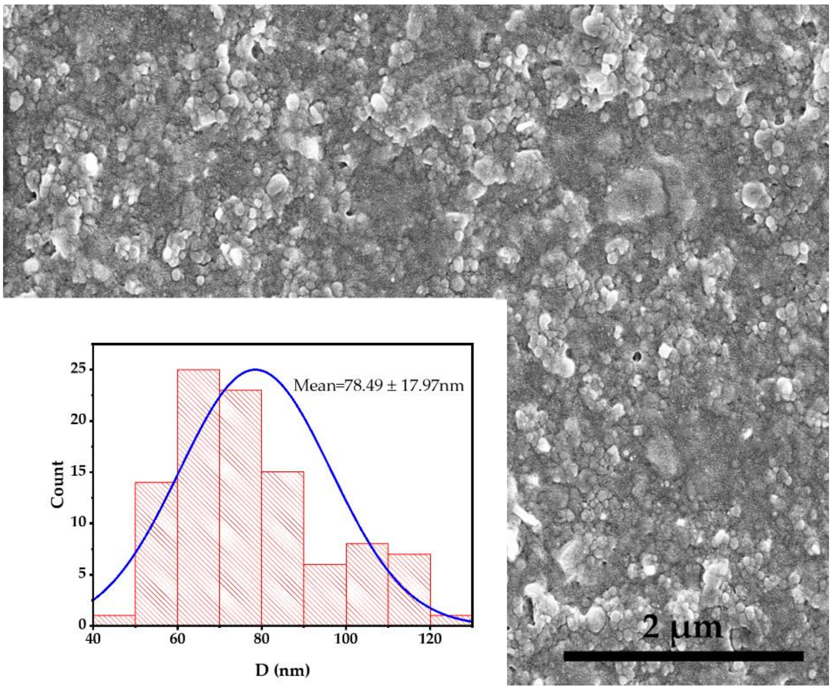

Figure S6. Scanning electron microscope (SEM) image and size distribution graph of $CsPbBr_3$ MCs obtained through ball milling process and ethanol/water treatment.
